# Cross Talk between Two Antioxidant Systems, Thioredoxin and DJ-1: Consequences for Cancer

**DOI:** 10.18632/oncoscience.12

**Published:** 2014-01-02

**Authors:** Prahlad V. Raninga, Giovanna Di Trapani, Kathryn F. Tonissen

**Affiliations:** ^1^ School of Biomolecular and Physical Sciences, Griffith University, Nathan, Qld, Australia; ^2^ Eskitis Institute for Drug Discovery, Griffith University, Nathan, Qld, Australia

**Keywords:** Thioredoxin, DJ-1, redox signalling, oxidative stress, antioxidants, cancer

## Abstract

Oxidative stress, which is associated with an increased concentration of reactive oxygen species (ROS), is involved in the pathogenesis of numerous diseases including cancer. In response to increased ROS levels, cellular antioxidant molecules such as thioredoxin, peroxiredoxins, glutaredoxins, DJ-1, and superoxide dismutases are upregulated to counteract the detrimental effect of ROS. However, cancer cells take advantage of upregulated antioxidant molecules for protection against ROS-induced cell damage. This review focuses on two antioxidant systems, Thioredoxin and DJ-1, which are upregulated in many human cancer types, correlating with tumour proliferation, survival, and chemo-resistance. Thus, both of these antioxidant molecules serve as potential molecular targets to treat cancer. However, targeting one of these antioxidants alone may not be an effective anti-cancer therapy. Both of these antioxidant molecules are interlinked and act on similar downstream targets such as NF-κβ, PTEN, and Nrf2 to exert cytoprotection. Inhibiting either thioredoxin or DJ-1 alone may allow the other antioxidant to activate downstream signalling cascades leading to tumour cell survival and proliferation. Targeting both thioredoxin and DJ-1 in conjunction may completely shut down the antioxidant defence system regulated by these molecules. This review focuses on the cross-talk between thioredoxin and DJ-1 and highlights the importance and consequences of targeting thioredoxin and DJ-1 together to develop an effective anti-cancer therapeutic strategy.

## INTRODUCTION

Cancer is currently one of the most deadly diseases worldwide. Amongst the many factors that cause cancer, oxidative stress is one of the most important and well-studied factors that give rise to the conditions leading to tumour development and progression [1]. Oxidative stress is associated with an increased concentration of reactive oxygen species (ROS). Depending on the cellular context, amount and exposure time, ROS can be either detrimental to the cells or important players in regulating various cellular responses including cell proliferation, differentiation, and apoptosis [2, 3]. It has been shown that low levels of ROS promote cell proliferation and differentiation by activating transcription factors, such as nuclear factor-κβ (NF-κβ) [4-6] and activator protein-1 (AP-1) [5-7]. On the other hand, excessive generation of cellular ROS results in oxidative stress, which induces cell death by caspase activation, activation of Bcl-2 family proteins, and modulation of protein kinases [8]. ROS-induced oxidative stress is involved in the pathogenesis of a wide-variety of diseases including neurodegenerative diseases, inflammation, cardiac disease, and cancer. Since an excess of ROS is detrimental to the cells or tissues, their detoxification is essential. Within the cells, there is a gambit of antioxidant molecules including thioredoxins [9, 10], glutaredoxins [11], peroxiredoxins [12] and other enzymes such as superoxide dismutase that detoxify ROS and maintain the balance between the generation and removal of oxidative species. During the past decade, DJ-1 has also emerged as an antioxidant molecule playing a crucial role in regulating cellular redox signalling cascades and inducing other antioxidants under oxidative stress conditions [13, 14]. Such antioxidant molecules sense physiological fluctuations and imbalance of intracellular redox state and respond to such imbalance by activating appropriate signalling cascades.

Despite the presence of such antioxidant defence systems within the cells, ROS generation often exceeds the antioxidant capacity of the cells leading to oxidative stress and ROS-induced cell death. In response to increased ROS levels, cells have evolved a number of survival pathways to counteract the toxic effect of ROS, which includes upregulation of antioxidant molecules and stress-response proteins. Upregulation of antioxidant molecules offer an advantage to the cells to eliminate the detrimental effects of ROS. However, cancer cells exploit this advantage and use it for their own protection against increased ROS levels. Antioxidant molecules have been shown to be upregulated in many human cancer types, correlating with tumour proliferation, survival, and drug resistance [15-19]. Inhibition of elevated antioxidants inhibits tumour growth and metastasis, promotes tumour apoptosis, and reverses tumour resistance to chemotherapy, further implicating the functionality of antioxidant systems in cancer development and progression [20-23]. Thus, such deregulated antioxidants may serve as potential molecular targets to develop new single or combinatory anti-cancer therapy.

In this review, we describe the role of DJ-1 and thioredoxin in cellular redox signalling and discuss the cross-talk between these two antioxidant systems to balance the cellular redox state. An understanding of the cross-talk between DJ-1 and Trx1 may help us in finding novel therapeutic targets for the treatment of many types of human cancers, where antioxidants are upregulated.

### DJ-1

DJ-1 was identified 16 years ago as a putative oncogene, transforming mouse NIH3T3 cells weakly on its own and strongly in combination with H-Ras [24]. Several studies have shown that the expression of DJ-1 is increased in several cancer types as compared to non-cancerous cells. High DJ-1 expression has been observed in primary lung and prostate cancer biopsies [25, 26], in non-small cell lung carcinoma patients [27] as well as in endometrial cancer patients [28]. Proteomic studies have identified DJ-1 as a secreted tumour antigen in breast cancer patients [29] and as a potential biomarker secreted in uveal malignant melanoma patients [30].

DJ-1 was first associated with neurodegeneration when a large deletion and missense mutation in the DJ-1 gene in Italian and Dutch Parkinson's disease patients was found, leading to identification of the DJ-1 gene associated with autosomal recessive early-onset Parkinson's disease [31]. Apart from the large genomic deletion within the DJ-1 coding region, there are other point mutations also responsible for the Parkinson's disease condition for example, L166P is responsible for severe destabilisation of the DJ-1 protein [31-36].

### Structure, Expression and Function of DJ-1

DJ-1 is a 189 amino acid protein dimer consisting of nine α-helics and seven β-strands [35-39]. The structure of the DJ-1 protein is similar to the monomer subunit of the intracellular cysteine protease from *Pyrococcushorikoshii*, protease 1 [35, 40], but DJ-1 contains an extra α-helix at the C-terminal region blocking the putative catalytic site of DJ-1 under normal conditions. This additional α-helix may undergo conformational changes under oxidative stress leading to the activation of DJ-1 catalytic site [35, 37].

DJ-1 is abundantly present in the majority of cells and tissues in the body [24], therefore there are no clues for cell- or tissue specific functions of DJ-1. DJ-1 is located in the cytosol, nucleus and mitochondria of the cells and it has also been reported to be secreted from the cells or tissues in cancer and Parkinson's disease patient's serum and from astrocytes [29, 41-44]. Upon exposure to growth factors and oxidative stress stimuli, DJ-1 translocates to the nucleus when cells are in the S phase of the cell cycle [24, 43].

DJ-1 has been implicated in many biological functions including transcriptional regulation [13, 45-47], chaperone activity regulation [48, 49], protease function regulation [42], and mitochondrial regulation [50, 51]. Of most significance, consistent findings demonstrate an antioxidant activity as well as a cytoprotective function against oxidative stress [52, 53] and a role in increasing cell survival under pro-apoptotic stimuli challenge [26]. Evidence for an antioxidant function of DJ-1 in neuronal cells comes from various studies. Rotenone and 6-hydroxydopamine-induced oxidative stress in neuroblastoma cells have upregulated endogenous DJ-1 protein and mRNA levels and induced translocation of DJ-1 from the cytoplasm to the mitochondria to exert neuroprotection function [54]. Overexpression of wild-type DJ-1, but not the mutant forms including L166P, C106S, M26I, and R98Q, protects neurons against ROS producing stress insult [55]. Several reports describe different mechanisms by which DJ-1 exerts its neuroprotective effects. DJ-1 protects primary dopaminergic neurons against hydrogen peroxide (H_2_O_2_) and 6-hydroxydopamine-induced oxidative stress by upregulating intracellular glutathione synthesis via increasing glutamate cysteine ligase enzyme [14]. DJ-1 also protects dopaminergic neurons from the toxic effects of mutant human α-synuclein by increasing heat shock protein 70 expression within the cells [14]. Taken together, these results suggest DJ-1 may exert neuroprotective action by different mechanisms, which may depend on the type of stress stimuli. In contrast to DJ-1 overexpression, siRNA mediated down-regulation of DJ-1 renders cells susceptible to undergo death under oxidative stress, ER stress, and proteasome inhibition [56], confirming the role of DJ-1 in protecting cells against oxidative stress.

The crystal structure of DJ-1 reveals the presence of three redox sensitive cysteine residues, Cys46, Cys53, and Cys106. Amongst these three cysteine residues, Cys106 is the most susceptible to oxidative stress and can be oxidized to SOH, SO_2_H, and then SO_3_H [50, 57]. Many studies have confirmed that DJ-1 requires Cys106 to exert its cytoprotection against oxidative stress [50, 58, 59], whereas only one study identifies Cys53 as the functionally active and essential residue [60]. Mutation of Cys106 abolishes all functions of DJ-1 [50, 53]. Mild oxidation of Cys106 to SO_2_H is critical for the cytoprotective function of DJ-1 [61, 62]. Oxidation of Cys106 to SO_2_H increases the mitochondrial localization of DJ-1 and allows the direct binding of DJ-1 to apoptosis signal-regulating kinase 1 (ASK1) to inhibit the activation of ASK1-mediated apoptosis [62, 63]. Further oxidation of Cys106 from SO_2_H to SO_3_H results in the aggregation of DJ-1, which leaves DJ-1 in its inactive form [48]. Analysis of the oxidation state of DJ-1 in post-mortem brain tissue of Parkinson's and Alzheimer's patients as well as in healthy individuals has revealed that DJ-1 from diseased tissue was extensively oxidized as compared to healthy cells [64]. In conclusion, the mild oxidized form of DJ-1 is associated with its cytoprotective action by binding to its target molecules and regulating their activity, whereas the highly oxidized form of DJ-1 is associated with diseased conditions.

### Role of DJ-1 in regulating transcription factors under oxidative stress

DJ-1 functions as an antioxidant when cells experience oxidative stress and acts to induce the expression of several antioxidant enzymes [14, 65]. For example, DJ-1 increases the expression of glutamate cysteine ligase, which is a rate-limiting enzyme for intracellular glutathione biosynthesis [65] while down-regulation of DJ-1 results in a decrease in expression of extracellular superoxide dismutase (SOD3) [14]. Under oxidative stress conditions, DJ-1 has been shown by many studies to regulate various transcription factors and transmit downstream signals to respond to oxidative stress. For example DJ-1 has been shown to regulate the sterol regulatory binding protein 2 (SREBP2), a transcription factor that regulates cholesterol synthesis [66]. Reporter gene assays were used to show that over expression of DJ-1 increased the promoter activity of the low-density lipoprotein receptor (LDLR) gene and this activity was further enhanced by oxidative stress [67]. By using chromatin immunoprecipitation (ChIP), gel-mobility shift and co-immunoprecipitation, it has been shown that DJ-1 forms a complex with SREBP2, which binds to the sterol regulatory element (SRE) in the LDLR gene promoter [67].

Several studies have identified DJ-1 as a positive regulator of another transcription factor, the androgen receptor (AR), either indirectly [45, 68] or directly [69]. PIAS-alpha, a protein inhibiting androgen receptor transcription activity, has been identified as a DJ-1 binding protein by a yeast two-hybrid screen [45]. DJ-1 has been shown to bind to the AR-binding region of PIAS-alpha *in vitro* by co-immunoprecipitation and *in vivo* in human 293T cells resulting in the inhibition of PIAS-alpha and therefore, activation of androgen receptor transcription activity [45]. Another study using a yeast two-hybrid screen has shown that DJ-1 can also directly bind to the androgen receptor [69].

Nuclear factor (erythroid-derived 2)-like 2 (Nrf2) is a redox-sensitive transcription factor, which serves as a master regulator of antioxidant and detoxifying genes under oxidative stress via the antioxidant response element (ARE) [70]. Under normal conditions, Nrf2 is localized in the cytoplasm and forms a complex with Kelch-like erythroid cell-derived protein-1 (Keap1) [71]. Keap1 is a negative regulator of Nrf2 as it targets Nrf2 for degradation by the ubiquitin-proteosome system [72]. Three cysteine residues have been identified in Keap1 that are involved in the regulation of Nrf2 [72]. Two of these cysteine residues are required for ubiquitination and degradation of Nrf2 in the cytoplasm whereas the third cysteine residue acts as a redox sensor, leading to conformational changes of Keap1 under oxidative stress [72]. These changes result in the prevention of Keap1-dependent inhibition of Nrf2 [72]. DJ-1 has been shown to stabilize the Nrf2 protein by preventing its association with Keap1. Co-immunoprecipitation experiments showed that DJ-1 overexpression eliminated the presence of Nrf2/Keap1 complexes, resulting in lower levels of Nrf2 ubiquitination [13]. Moreover, it has been reported that DJ-1 is required for Nrf2-regulated transcription of its target genes since siRNA-induced inhibition of DJ-1 resulted in the reduction of NQO1 ARE luciferase activity even when cells are treated with the classical Nrf2 inducer, tert-butylhydroquinone (tBHQ) [13]. ChIP experiments failed to detect DJ-1 on the promoter, indicating it does not bind with the Nrf-2 complex to AREs. Although experimental data suggests the role of DJ-1 in stabilizing Nrf2 is by inhibiting Nrf2/Keap1 complex formation, there is no experimental evidence showing direct interaction of DJ-1 with either Nrf2 or Keap1 and therefore the exact mechanism remains elusive [13]. In contrast, another study has reported that activation of the Nrf2-ARE pathway is independent of DJ-1 [73]. It was shown that tBHQ can still activate the Nrf2-ARE pathway and protect primary cortical neurons derived from DJ-1-knockout as well as DJ-1 wild-type mice. This indicates that DJ-1 is not required for Nrf2-regulated transcription of its target gene and activation of the Nrf2-ARE pathway [73]. Hence, the question of the functional effect of DJ-1 on Nrf2 activity still remains open with the possibility of redundant activation pathways.

Another redox sensitive transcription factor regulated by DJ-1 is p53. p53 exerts many cellular functions including induction of senescence, apoptosis and regulating mitochondrial homeostasis against oxidative stress. Using luciferase assays, it has been shown that Topors represses the transcriptional activity of p53 and that its activity is stimulated by SUMO-1 [46]. This result suggests that p53 activity is inhibited by Topors-mediated sumoylation of p53. Using co-immunoprecipitation assays, DJ-1 was shown to bind to p53 *in vivo* as well as *in vitro.* Using luciferase assays and co-immunoprecipitation experiments, DJ-1 was shown to restore the transcriptional activity of p53 through SUMO-1 conjugation suggesting that DJ-1 may act as a positive regulator of p53 [46]. Conversely, other studies have shown that DJ-1 inhibits the transcriptional activity of p53. Co-immunoprecipitation studies have shown that DJ-1 physically interacts with p53 *in vitro* as well as *in vivo* resulting in the inhibition of p53 transcription activity [74]. DJ-1-mediated repression of p53 transcription activity leads to the down-regulation of Bax and suppression of caspase cleavage [74]. Another study has reported that the oxidized form of DJ-1 binds to the DNA-binding region of p53 and inhibits p53 transcription activity when DNA-binding affinity of p53 is low [75]. Whether DJ-1 acts as a positive or negative regulator of p53 transcriptional activity may depend on the extent of oxidation of DJ-1.

**Figure 1:**
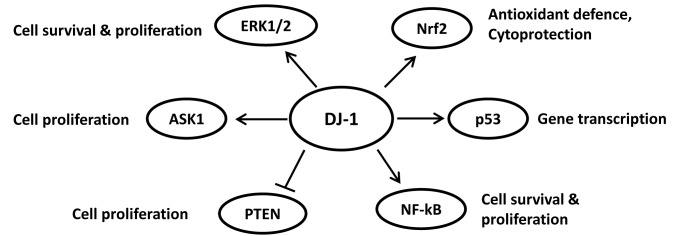
Summary of DJ-1 functions by regulating redox sensitive transcription factors and signalling pathways

Another important transcription factor regulated by DJ-1 is nuclear factor-κβ (NF-κB). DJ-1 directly interacts with one of its binding partners, Cezanne, which is a physiological inhibitor of the transcription activity of NF-κβ [76]. The interaction of DJ-1 with Cezanne was shown by mass spectrometry and co-immunoprecipitation. This interaction inhibits the deubiquitination activity of Cezanne allowing the activation of NF-κβ transcription activity. Thus, DJ-1 has been reported to be a positive regulator of NF-κβ, which highlights another potential mechanism by which DJ-1 promotes cell survival in cancer [76]. A summary of the transcription factors regulated by DJ and its downstream functions are described in Figure 1.

Taken all together, these findings support the suggestion that DJ-1 might be a master upstream regulator of various transcription factors and pathways regulating various cellular responses including cell proliferation and apoptosis. Therefore, DJ-1 can serve as a potential therapeutic target for different cancer types where such transcription factors are deregulated.

### DJ-1-Induced Signal Transduction under Oxidative Stress

In addition to the roles of DJ-1 in the regulation of various transcription factors, many studies have reported an involvement of DJ-1 in various signalling pathways and interactions with different signalling molecules. Several studies have reported the role of DJ-1 in regulating the tumour suppressor protein, phosphatase and tensin homolog (PTEN) [27, 59, 77]. Using a genetic screen of *Drosophila* gain-of-function mutants, DJ-1 was shown to be a negative regulator of PTEN functions, resulting in the up-regulation of the phophoinositide3-kinase (PI3K)/Akt signalling pathway [27]. The PI3K/Akt pathway is a redox sensitive growth signalling pathway [78]. Once activated, PI3K causes phosphorylation of Akt/protein kinase B (PKB), which stimulates cell growth. PTEN inhibits PI3K and negatively regulates the PI3K/Akt signalling pathway. Over-expression of DJ-1 leads to increased phosphorylation of P13K targets, leading to increased cell survival [27]. H_2_O_2_-induced oxidative stress inactivates PTEN leading to the activation of downstream cell growth signalling molecules including Akt/protein kinase B [78]. Using co-immunoprecipitation and pull-down assays, wild-type and C106S mutant DJ-1 were shown to interact directly with PTEN in mouse NIN3T3 cells [59]. Under oxidative stress, oxidized DJ-1 directly binds to PTEN, decreases its phosphatase activity and increases phosphorylation of AKT, resulting in the transmission of cell survival signals in NIH3T3 cells [59]. Thus, DJ-1 activates a cell growth signalling cascade by inhibiting PTEN and resulting in activation of the PI3K signalling pathway. It can be hypothesized that inhibition of DJ-1 may prevent PI3K-regulated cell proliferation signalling pathways. This may result in a new therapeutic regime for the treatment of cancers where DJ-1 is over-expressed and leads to the activation of the PI3K/Akt/PKB signalling axis.

DJ-1 exerts cytoprotective action against ultraviolet (UV)-induced oxidative stress by directly interacting with mitogen-activated protein kinase/extracellular signal-regulated kinase kinase kinase 1 (MEKK1), suppressing MEKK1 activity and acting as a negative regulator of the JNK signalling cascade to suppress cell death [79]. The extracellular signal-regulated kinase (ERK) is the main pathway leading to cell-migration and is essential for neuroprotection. The ERK1/2 signalling cascades can be modified when the redox state of the cells changes in Parkinson's disease. It was reported that over-expression of wild-type DJ-1 enhanced the phosphorylation of ERK1/2 and upstream kinase MEK1/2 [80]. DJ-1-induced activation of the ERK signalling pathway resulted in increased cell viability and cytoprotection against H_2_O_2_ [80]. Conversely, another study has reported that DJ-1 is up-regulated by the activation of the MAP kinase pathway via increased ERK1/2 phosphorylation in human neuroblastoma cells exposed to dopamine [52]. This conclusion was made using MAPKK inhibitors, which prevented the dopamine induced DJ-1 up-regulation. However, a more recent study showed DJ-1 could protect dopaminergic neurons against rotenone-induced apoptosis by increasing phosphorylation of ERK and thus inducing mitophagy [81]. Thus, mounting evidence suggests that DJ-1 may exert its cytoprotective function through the ERK signalling pathway to promote cell survival under oxidative stress.

Stable overexpression of DJ-1 protects cardiac cells against oxidative stress generated by increased ROS levels under hypoxic conditions [82]. The authors showed that overexpression of DJ-1 in cardiac cells reduces ischemia/reperfusion (sI/R)-induced ROS generation, up-regulates the expression of antioxidant enzymes, and prevents sI/R-induced oxidative stress [82]. The results from these cardiac cell studies suggest that DJ-1 has a potential role in inhibiting hypoxia-induced cell death and thus, emerges as a possible novel therapeutic target for the treatment of hypoxic tumour cells.

**Figure 2:**
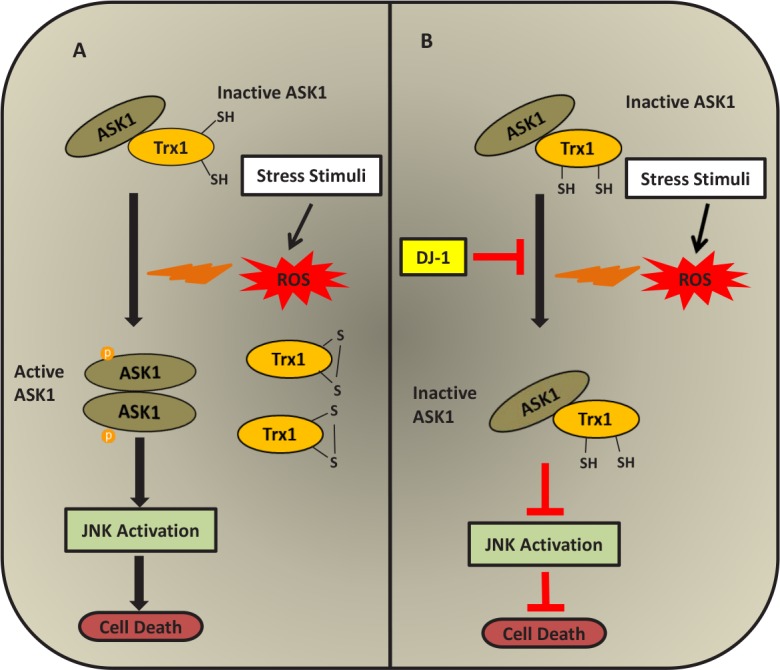
Regulation of ASK1/Trx1 by DJ-1 under oxidative stress A. In the absence of DJ-1, ASK1 is dissociated from Trx1 under oxidative stress conditions and gets activated. Activated ASK1 activates JNK and results in cell death. B. Over-expression of DJ-1 inhibits dissociation of ASK1 from Trx1 under oxidative stress and thus, inhibits ASK1 activation and exerts cytoprotection.

Another important signalling pathway that gets activated under oxidative stress is the Apoptosis Signal-regulating Kinase 1 (ASK1) pathway. ASK1 is an important component in activating the apoptosis signalling machinery induced by ROS generating cytotoxic stress [83, 84]. Oxidative stress induced by H_2_O_2_ causes dimerization of ASK1 and leads to its activation [85]. There are many studies showing the involvement of DJ-1 with ASK1 signalling cascades under oxidative stress [58, 63, 86-88]. Under oxidative stress conditions, Death-associated protein 6 (Daxx) translocates from the nucleus to the cytoplasm and interacts directly with ASK1, resulting in activation of the ASK1-mediated apoptotic signalling pathway [86]. Other studies have shown that wild-type DJ-1 exerts a cytoprotective action by sequestering Daxx in the nucleus in H_2_O_2_-treated cultured mammalian cells as well as in MPTP-treated Parkinson's disease model mice [87, 88]. Sequestration of Daxx by DJ-1 impedes its translocation into the cytoplasm, resulting in the prevention of ASK1/Daxx complex formation such that activation of apoptotic signal transmission does not occur. DJ-1 also binds directly to ASK1 under oxidative stress and inhibits its kinase activity, resulting in the inhibition of p38 mitogen-activated protein kinase (MAPK) signalling cascade and preventing cell death signals [63]. Under basal conditions ASK1 is bound to and inhibited by a physiological inhibitor, Thioredoxin1 (Trx1). Upon oxidative stress, Trx1 dissociates from ASK1 and thus ASK1 is activated, resulting in the transmission of apoptosis-inducing signals through its kinase activity [89]. DJ-1 has been shown to be a regulator of the ASK1/Trx1 interaction. Wild-type DJ-1 has been reported to prevent the dissociation of ASK1 from Trx1 under H_2_O_2_-induced oxidative stress in a cultured mammalian cell line as well as in mice whole brain homogenates using co-immunoprecipitation experiments [58]. DJ-1, by hindering ASK1/Trx1 dissociation, has been shown to supresses JNK activation and thus prevents H_2_O_2_-induced cell death [58]. Thus, it is interesting to note that DJ-1 and thioredoxin, known for antioxidant functions, are linked together and play a role in cytoprotection. A schematic representation of the interaction of ASK-1 and Trx and the regulation of this complex by DJ-1 under oxidative stress is illustrated in Figure 2.

Thus, DJ-1 is involved in multiple redox signalling pathways including the DJ-1/ASK1/Trx1 axis, which transmit cell proliferation signals. This indicates that targeted inhibition of DJ-1 may help in deactivating redox-responsive signalling events, altering the antioxidant activity of Trx1 and other antioxidant molecules to stimulate cell death in cancer and other diseases. Figure 1 summarises the action of DJ-1 on various signalling molecules and its function.

### The Thioredoxin System

The thioredoxin system is one of the most important antioxidant system present in all species [9, 10]. The thioredoxin system is comprised of thioredoxin reductase (TrxR) enzyme, NADPH, and thioredoxin (Trx). Trx has a conserved Cys-Gly-Pro-Cys redox catalytic site, which reduces the target proteins [9]. TrxR transfers reducing equivalents from NADPH to Trx and reduces the active site disulfide of oxidized Trx (Trx-S_2_) to a dithiol (Trx-(SH)_2_). The mammalian thioredoxin system is quite complex and both Trx and TrxR are expressed as different isoforms; Trx as Trx1 and Trx2, and TrxR as TrxR1 and TrxR2. Trx1 and TrxR1 are predominantly expressed in the cytosol, whereas Trx2 and TrxR2 are expressed in the mitochondria. All four of these genes are essential, since knockout mice lacking any of these four genes die early, during embryogenesis [90-93]. Another mammalian TrxR has also been identified, predominantly expressed in testis, which has the potential to reduce not only Trx but also glutathione disulfide. Therefore, this TrxR isoform is termed the thioredoxin/glutathione reductase (TGR) [94]. Amongst all the members of thioredoxin system, Trx1 and TrxR1 have emerged as critical redox regulators and as potential therapeutic targets for many human cancer types [17, 20, 95].

### Regulation of thioredoxin system under oxidative stress

Under oxidative stress conditions, the induction of thioredoxin expression is regulated by an ARE present in the promoter region of the Trx1 gene, which is bound by Nrf2 [96]. Upon activation, Nrf2 is translocated to the nucleus where it forms a heterodimer with maf proteins. Several studies have reported that theNrf2/Maf complex then binds to the ARE element in the promoter region of the target antioxidant genes [97] including thioredoxin [96, 98] and thioredoxin reductase [99] and increases their expression.

### Role of Trx in intracellular redox signalling pathways

Being critical redox regulators, Trx and TrxR are involved in a number of intracellular redox signalling pathways by activating a number of redox-sensitive transcription factors required for various cellular processes.

**Figure 3:**
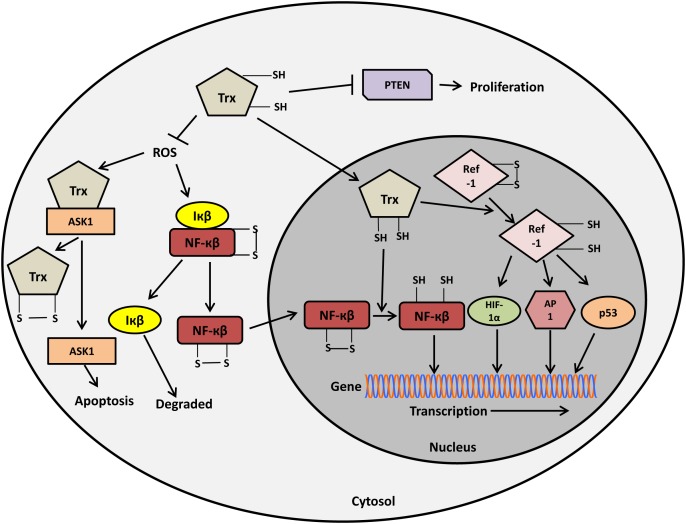
Role of thioredoxin in regulating redox sensitive transcription factors and signalling pathways Figure adapted and used with permission from the authors [135].

**Figure 4:**
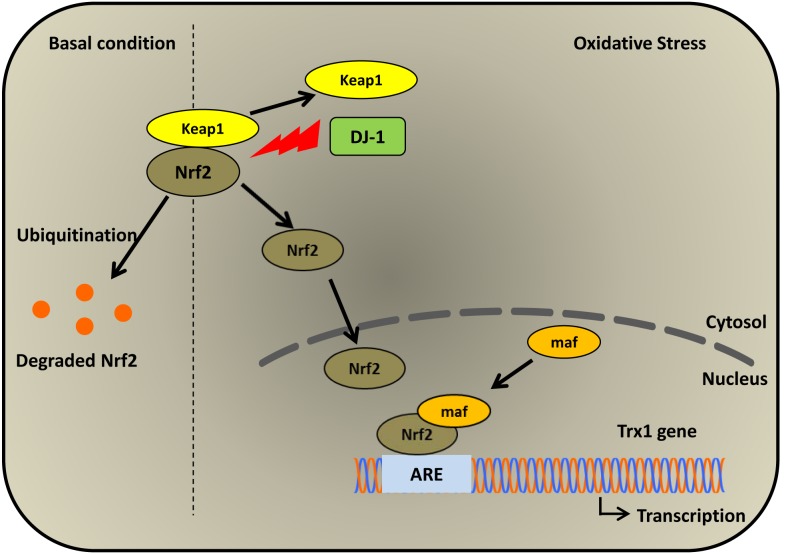
Induction of Trx1 expression by DJ-1 via the Nrf2 pathway Under oxidative stress, DJ-1 inhibits the interaction between Nrf2 and its inhibitor Keap1 resulting in activation of Nrf2. Activated Nrf2 translocates to the nucleus and binds the ARE element in the Trx1 promoter and induces Trx1 expression. Figure adapted and used with permission from the authors [135].

Trx is usually present in the cytoplasm, but under certain circumstances it is translocated to the nucleus. In the nucleus, Trx has been shown to activate redox sensitive transcription factors, including NF-κβ [100]. Under normal conditions, NF-κβ is present in the cytoplasm where it is inhibited and sequestered by its physiological inhibitor, inhibitor of-κβ (Iκβ). Under oxidative stress, ROS releases Iκβ subunit from NF-κβ which is then translocated to the nucleus [101]. In the nucleus, Trx reduces Cys62 in the NF-κβ subunit p50 allowing NF-κβ to bind to the recognition site present on the promoter region of its target genes, which are involved in cellular processes including, cell growth and survival (Figure 3) [100-102]. Trx also regulates the activity of other transcription factors such as Activator protein-1 (AP-1), via Redox factor-1 (Ref-1). Trx reduces Ref-1 in the nucleus, which in turn reduces cysteine residues present in the DNA-binding domains of Fos and Jun and thus activates AP-1 (Figure 4) [103]. Using *in vitro* diamide-induced cross-linking study and *in vivo* mammalian two-hybrid assays, Trx1 was shown to directly bind to Ref-1 in the nucleus and to activate the transcriptional activity of AP-1 [104].

Several studies have reported the involvement of Trx1 in regulating the activity of p53 [105-108]. By using electrophoretic mobility shift assays, it was shown that Trx enhanced the sequence-specific DNA binding activity of p53 in a Ref-1 dependent as well as independent manner [105]. Using luciferase assays, Trx was shown to stimulate the Ref-1 mediated transactivation of p53, which suggests an importance of functional coupling between Trx and Ref-1 in activating the p53 signalling cascade [105]. Furthermore, Trx has also been shown to be positively associated with p53-induced DNA repair in breast cancer cells [106].

Trx1 has been implicated in the regulation of the tumour suppressor activity of PTEN [109, 110], but there is a discrepancy in the data. PTEN is a redox sensitive tumour suppressor, which is inactivated by H_2_O_2_-induced ROS generation. PTEN exposure to ROS results in the formation of an intramolecular disulphide bond involving a redox sensitive cysteine residue (Cys124) present in the active site of PTEN, which inhibits PTEN tumour suppressor activity [109]. As described earlier, inhibition of PTEN results in the activation of Akt/protein kinase B and promotes cell survival. Using co-immunoprecipitation assays, Trx was shown to bind to an inactive oxidized form of PTEN and to reduce it, resulting in the activation of PTEN activity [109]. Another study used molecular docking studies and site-directed mutagenesis to also show that the reduced form of Trx1 directly binds to the C2 domain of PTEN by forming a disulphide bond between the Cys32 of Trx1 and Cys124 of PTEN [110]. In contrast to the previous study this binding of Trx1 to PTEN results in the inactivation of the lipid phosphatase activity and membrane binding of PTEN (Figure 3) [110]. Hence, Trx1 has been reported to impede PTEN activity as well as to stimulate PTEN functions. While the exact role of Trx1 in regulating PTEN activity remains a topic of debate, a recent study has shown that Trx1 inhibits PTEN activity in neuroblastoma cells [111]. Thus, Trx1, by inhibiting PTEN activity, may result in tumour cell proliferation. Taken all together, the Trx1/PTEN axis may be considered as an effective molecular target for the treatment of cancers with higher Trx1 levels and lowered PTEN activity.

Another target of Trx is ASK1 [89]. In the cytoplasm Trx1 has been shown to directly bind to Cys-250 in the N-terminal region of ASK1, causing inhibition of its kinase activity [112]. In the mitochondria Trx2 has been shown to directly bind to Cys-30 in the N-terminal region of ASK1, resulting in the inhibition of ASK1 activity [112]. Thus, ASK1 is regulated by both cytosolic and mitochondrial thioredoxin in an independent manner. ASK1 is activated by a variety of external stress stimuli including ROS. Under normal conditions the reduced form of Trx1 [Trx1-(SH)_2_] binds to ASK1, but under oxidative stress conditions the reduced form of Trx1 is converted to its oxidized form (Trx1-S_2_) and results in the dissociation of Trx1 from ASK1 [89, 112, 113]. This dissociation results in the activation of ASK1 kinase activity, which stimulates the apoptotic signalling cascade in the cells (Figure 2A).

The ERK pathway is also redox regulated by Trx. Recent evidence suggests that the loss of Trx1 or TrxR1 sensitised a mouse mammary carcinoma cell line (EMT6) to tumour necrosis factor – α (TNFα)-induced apoptosis by increasing nuclear localization of pERK-1/2 in a PI3K dependent manner [114]. This may suggest that increased Trx1 or TrxR1 levels in cancer cells prevents nuclear translocation of pERK-1/2 and therefore protects cells against TNFα-mediated apoptosis. Interestingly, cytoplasmic and mitochondrial localization of pERK-1/2 in Trx1 or TrxR1-deficient EMT6 cells have not sensitised cells to TNFα-induced apoptosis. These results indicate that the subcellular localization of pERK-1/2 is a critical determinant of whether cancer cells with compromised Trx levels or activity will undergo stress stimuli-induced apoptosis [114].

Thus, Trx1 is involved in multiple redox-regulated signalling pathways in cancer by regulating redox-sensitive transcription factors and signalling molecules (Figure 3). Taken together, modulation of Trx1 expression and activity in diseased cells leads to the modulation of the signal transmission regulated by various transcription factors and may emerge as an effective therapeutic approach to overcome cancer.

### Cross-talk between two antioxidant molecules: DJ-1 and Thioredoxin

Oxidative stress is one of the major factors giving rise to diseased conditions. As discussed above, DJ-1 and Trx are two important antioxidant molecules that play crucial roles in maintaining the intracellular redox state and provide cytoprotection against oxidative stress. DJ-1 has been shown to exert cytoprotection against oxidative stress in Parkinson's disease [14, 52-55]. DJ-1 protein levels are increased in a number of human cancer types as compared to normal cells or tissues and promote cell survival and resistance against apoptosis [25-30]. Similarly, Trx1 protein levels are significantly upregulated in many human cancer cell types including gastric, lung, colon, liver, and pancreatic cancers [17, 115-117] to provide protection against ROS-mediated cell death.

Both DJ-1 and Trx1 were considered as two separate intracellular antioxidant systems until two independent studies reported the interaction between DJ-1 and Trx1 [58, 118]. DJ-1 has been shown to enhance Trx1 expression by activating the transcriptional activity of Nrf2 *in vitro* as well as *in vivo* (Figure 4) [118]. Overexpression of wild-type DJ-1 induced the expression of Trx1 protein in HeLa cells, suggesting that DJ-1 may regulate the expression of Trx1 [118]. This result was further confirmed when inhibition of DJ-1 expression in neuroblastoma cells and DJ-1 knockout in mice significantly reduced Trx1 protein and mRNA levels [118]. Overexpression of DJ-1 enhanced Nrf2 protein levels and increased its nuclear translocation, which was confirmed by western blot analysis [118]. Using promoter assay and chromatin co-immunoprecipitation (ChIP) assays, it was shown that DJ-1 enhanced the recruitment of Nrf2 on the ARE region of the Trx1 promoter and increased Trx1 mRNA expression [118]. However DJ-1 was not bound to the ARE. Thus DJ-1 may exert its cytoprotective effect against oxidative stress by inducing expression of Trx1 via Nrf2 [118]. Figure 4 illustrates the action of DJ-1 on Trx1 expression. It was shown that the treatment of cells with 1-chloro-2, 4-dinitrobenzene, a pharmacological inhibitor of TrxR, and Trx1 specific siRNA resulted in the partial loss of DJ-1-mediated cytoprotection against oxidative stress [118]. Although DJ-1 was reported to stimulate the transcriptional activity of Nrf2, the exact mechanism still remains elusive. DJ-1 has been shown to increase Nrf2 protein levels without affecting Nrf2 mRNA expression by preventing the interaction between Nrf2 and Keap1 [13, 119]. By using co-immunoprecipitation experiments it was shown that Keap1 was bound to Nrf2, but the presence of DJ-1 eliminated this association in Huh7 cells [13]. This data demonstrates that DJ-1 hinders the association between Nrf2 and Keap1, however it does not prove the physical interaction of DJ-1 with either Nrf2 or Keap1 [13]. In contrast, another study, by using co-immunoprecipitation and chemical cross-linking experiments, has shown that DJ-1 neither physically interacts with Nrf2 or Keap1 nor interferes with the interaction between Nrf2 and Keap1 [118]. Thus, a question of how DJ-1 activates Nrf2 is still open and seeks more understanding of the mechanism. These findings suggest that, Trx1 and DJ-1 are not only independent antioxidant molecules but DJ-1 may serve as an upstream regulator of Trx1, at least for some stress conditions.

Apart from the DJ-1/Trx1 interaction, there are no reports describing any role for DJ-1 in regulating expression and activity of other members of the thioredoxin system, including Trx2. Trx2 is a mitochondrial thioredoxin and exerts cytoprotection against external stress stimuli. Trx2 has been shown to bind mitochondrial ASK1 and inhibits mitochondrial ASK1-mediated apoptosis in a JNK-independent fashion [112]. Based on the observations that DJ-1 regulates the cytosolic ASK1/Trx1 complex to inhibit ASK1-mediated apoptosis [58] and its mitochondrial localization [50, 51] to exert neuroprotection, it would be interesting to determine if DJ-1 also regulates the mitochondrial Trx2/ASK1 complex. It is possible that the DJ-1/Trx2/ASK1 axis may serve as a novel molecular mechanism to protect cells against mitochondrial ROS-induced apoptosis. An understanding of these molecular signalling pathways may open new avenues for the therapeutic intervention of cancers.

### Therapeutic perspective

The intracellular redox state regulates various cellular signalling pathways under oxidative stress conditions induced by internal and external stimuli. DJ-1 and Trx play a crucial role in maintaining intracellular redox homeostasis by involving and regulating various redox stress responsive signalling pathways. Thus, targeting either of these antioxidant molecules or both together, may serve as a novel and effective therapeutical approach for the treatment of cancers that acquire resistance to the anti-cancer therapy acting by modulating ROS levels. DJ-1 and Trx play a protective role against oxidative stress and inhibit redox stress-induced cell death in many diseases. Genetic mutations of DJ-1 resulting in the loss or reduced DJ-1 functions lead to the onset of oxidative stress-related diseases including Parkinson's disease [31, 120, 121], stroke [122], chronic obstructive pulmonary disease (COPD) [119], and type II diabetes [123]. Expression of DJ-1 has been shown to be increased in many cancer types [25-27, 29, 77]. Therefore, the knowledge about the antioxidant and cytoprotective functions of DJ-1 obtained from other disease models can be readily translated into cancers where an expression of DJ-1 is upregulated. Similarly, Trx has been shown to act as a modulator of cell susceptibility under oxidative stress conditions and exerts protective effects on cancer cells [124]. Trx1 expression has been reported to increase in many cancer cell types [17, 114-117] where it results in increased cell proliferation and resistance to cell death [116]. The thioredoxin system has emerged as a potential therapeutic target for the treatment of many human cancer types using compounds that specifically target the thioredoxin system to induce cancer cell apoptosis [20, 95]. Inhibition of Trx1 or TrxR1 using specific inhibitors inhibits tumour cell proliferation, induces apoptosis, and increases the sensitivity of cancer cells to anti-cancer therapy [20, 21].

Findings that have implicated the role of DJ-1 in inducing Trx1 expression by increasing Nrf2 activity, have given another mechanism by which DJ-1 exerts cytoprotective function. Inhibition of Trx1 expression by Trx1-specific siRNA has decreased the DJ-1 induced cytoprotective effect against oxidative stress [118], but has not completely eliminated it. DJ-1 may increase expression and activity of other Nrf2-regulated antioxidant or detoxifying enzymes including heme oxygenase 1 (HO-1) and NAD(P)H quinine oxidoreductase 1 (NQO1), which may protect cells against oxidative stress rendering therapies targeting Trx1 alone less effective. Furthermore, targeting DJ-1 alone may not be sufficient to increase cell susceptibility to ROS-induced apoptosis because the Nrf2-regulated signalling cascade can also be activated by regulatory molecules other than DJ-1 such as the autophagy substrate p62, p53-targeted p21 protein [125, 126]. Therefore, inhibition of DJ-1 or Trx1 alone may not be sufficient to treat cancers with high antioxidant levels. Targeting the DJ-1/Nrf2/Trx1 axis may be important in modulating cellular response to ROS-induced cell death in cancer. Furthermore, elucidation of the possible interactions of DJ-1 with the other members of Trx system may also lead to the identification of novel molecular therapeutic targets that can be used to develop anti-cancer therapies.

Another molecular axis that may raise a significant interest for the treatment of various types of malignancies is the Trx1/DJ-1/PTEN axis. Tumour suppressor PTEN is reported to be lost or inhibited in many types of human cancers [127-131] and is deregulated in many other diseased conditions including, rheumatoid arthritis, chronic pulmonary disease, and pulmonary fibrosis [132-134]. Since both Trx1 and DJ-1 target the tumour suppressor activity of PTEN, co-inhibition of both of these antioxidant molecules may prove an effective approach for the therapeutic intervention of chemotherapy-resistant forms of cancer.

NF-κβ is a redox-sensitive transcription factor that is positively regulated by both DJ-1 and Trx1 [76, 100]. As discussed earlier, DJ-1 activates NF-κβ by binding to its physiological inhibitor, Cezanne [76], whereas Trx binds directly to the NF-κβ in the nucleus and reduces a key cysteine residue to enhance the DNA binding activity of NF-κβ [100]. Therefore, there are two distinct mechanisms that up-regulates NF-κβ activity. Thus, the DJ-1/NF-κβ/Trx1 axis is another molecular axis that may be considered as a potential target to treat cancer where these antioxidants are upregulated.

**Figure 5:**
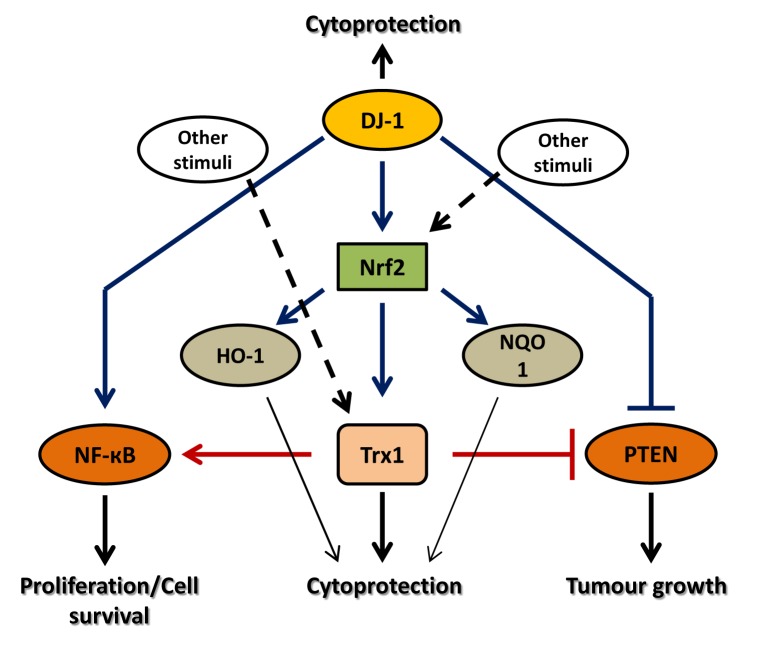
Consequences of targeting each antioxidant, Trx1 and DJ-1, alone or in combination in cancer Both Trx1 and DJ-1 exert cytoprotection by independent mechanisms as well as by acting on common targets such as, NF-κB and PTEN. Targeting DJ-1 alone may not be sufficient to induce cell death since Nrf2 can also be activated by other stress stimuli leading to Trx1 upregulation. Moreover, Trx1 also activates NF-κB and inhibits the tumour suppressor activity of PTEN leading to cell survival and tumour growth. Thus even after inhibition of DJ-1, all other cytoprotective machineries are functional and may promote tumour growth. Similarly, targeting Trx1 alone may not induce cancer cell death since DJ-1 may activate other Nrf2-targeted cytoprotective genes, or activates NF-κB, or inhibits tumour suppressor activity of PTEN resulting in cell survival and tumour growth. On the other hand, targeting Trx1 and DJ-1 together may completely shut down all the cytoprotective machineries regulated by these antioxidants and leads to cancer cell apoptosis. Hence, targeting two or more antioxidants in conjunction may prove an effective therapy to treat cancer.

Thus, tumours having elevated levels of antioxidants, such as Trx1 and DJ-1, may not respond well to the therapies targeting only one of them. Inhibiting either Trx1 or DJ-1 alone may allow the other to activate downstream signalling cascade leading to the tumour cell survival and proliferation. On the other hand, targeting both Trx1 and DJ-1 in conjunction may completely shut down the antioxidant defence systems regulated by these antioxidants and render the cancer cells sensitive to ROS-induced cell death. Figure 5 summarises the consequences of targeting Trx1 and DJ-1 alone or in combination in cancer cells.

## CONCLUSIONS

Although the precise mechanisms of the pathogenesis of cancers occurring due to redox stress is not fully resolved, dysregulation of intracellular redox state and redox-regulated signalling cascades are responsible for the alterations in the normal cellular functions. Full elucidation of DJ-1 and Trx-dependent regulation of redox signalling cascades may provide an effective and a promising therapeutic regime to treat cancers with high antioxidant levels. Furthermore, elucidation of the cross-talk between DJ-1 and other members of the Trx superfamily in different tumour models, and the molecular mechanism of these interactions may lead to the identification of multiple molecular targets. Detailed understanding of the role of DJ-1 in regulating Nrf2-mediated signalling pathways and its targeted genes may lead to the identification of novel targets for therapeutic intervention of various human cancer types.
